# The TRAIL in the Treatment of Human Cancer: An Update on Clinical Trials

**DOI:** 10.3389/fmolb.2021.628332

**Published:** 2021-03-10

**Authors:** Martin Snajdauf, Klara Havlova, Jiri Vachtenheim, Andrej Ozaniak, Robert Lischke, Jirina Bartunkova, Daniel Smrz, Zuzana Strizova

**Affiliations:** ^1^ Third Department of Surgery, First Faculty of Medicine, Charles University and University Hospital Motol, Prague, Czechia; ^2^ Department of Urology, Second Faculty of Medicine, Charles University and University Hospital Motol, Prague, Czechia; ^3^ Department of Immunology, Second Faculty of Medicine, Charles University and University Hospital Motol, Prague, Czechia

**Keywords:** TRAIL clinical trials, recombinant TRAIL, TRAIL-receptor agonists, cancer, dulanermin, mapatumumab

## Abstract

TRAIL (tumor-necrosis factor related apoptosis-inducing ligand, CD253) and its death receptors TRAIL-R1 and TRAIL-R2 selectively trigger the apoptotic cell death in tumor cells. For that reason, TRAIL has been extensively studied as a target of cancer therapy. In spite of the promising preclinical observations, the TRAIL–based therapies in humans have certain limitations. The two main therapeutic approaches are based on either an administration of TRAIL-receptor (TRAIL-R) agonists or a recombinant TRAIL. These approaches, however, seem to elicit a limited therapeutic efficacy, and only a few drugs have entered the phase II clinical trials. To deliver TRAIL-based therapies with higher anti-tumor potential several novel TRAIL-derivates and modifications have been designed. These novel drugs are, however, mostly preclinical, and many problems continue to be unraveled. We have reviewed the current status of all TRAIL-based monotherapies and combination therapies that have reached phase II and phase III clinical trials in humans. We have also aimed to introduce all novel approaches of TRAIL utilization in cancer treatment and discussed the most promising drugs which are likely to enter clinical trials in humans. To date, different strategies were introduced in order to activate anti-tumor immune responses with the aim of achieving the highest efficacy and minimal toxicity.In this review, we discuss the most promising TRAIL-based clinical trials and their therapeutic strategies.

## Introduction

Several therapeutic strategies are currently subjected to testing with the aim to trigger the antitumor immune responses (Zhang and Zhang, 2020; Nagai and Kim, 2017). TRAIL (tumor—necrosis factor related apoptosis-inducing ligand, CD253) is a protein initially described to selectively trigger extrinsic apoptotic pathway in malignant cells (Micheau et al., 2013). Moreover, TRAIL engages the apoptosis of tumor cells in a p53 independent manner, unlike most of chemotherapeutic drugs (Micheau et al., 2013; Willms et al., 2019). TRAIL has a sequence homology to TNF (tumor necrosis factor) and Fas ligand and, as a member of the tumor necrosis factor superfamily, binds to death receptors DR4 (TRAIL-R1) and DR5 (TRAIL-R2), decoy receptors DcR1 and DcR2, and osteoprotegerin (OPG) (Yuan et al., 2018). Death receptors DR4 and DR5 bear agonistic properties due to their intracellular death domain (DD) which transmits the apoptotic signal. DcR1, DcR2 and OPG, on the other hand, lack DD and, therefore, act as regulatory receptors. TRAIL is naturally found as a trimer which induces oligomerization of its receptors (Dubuisson and Micheau, 2017).

**FIGURE 1:**
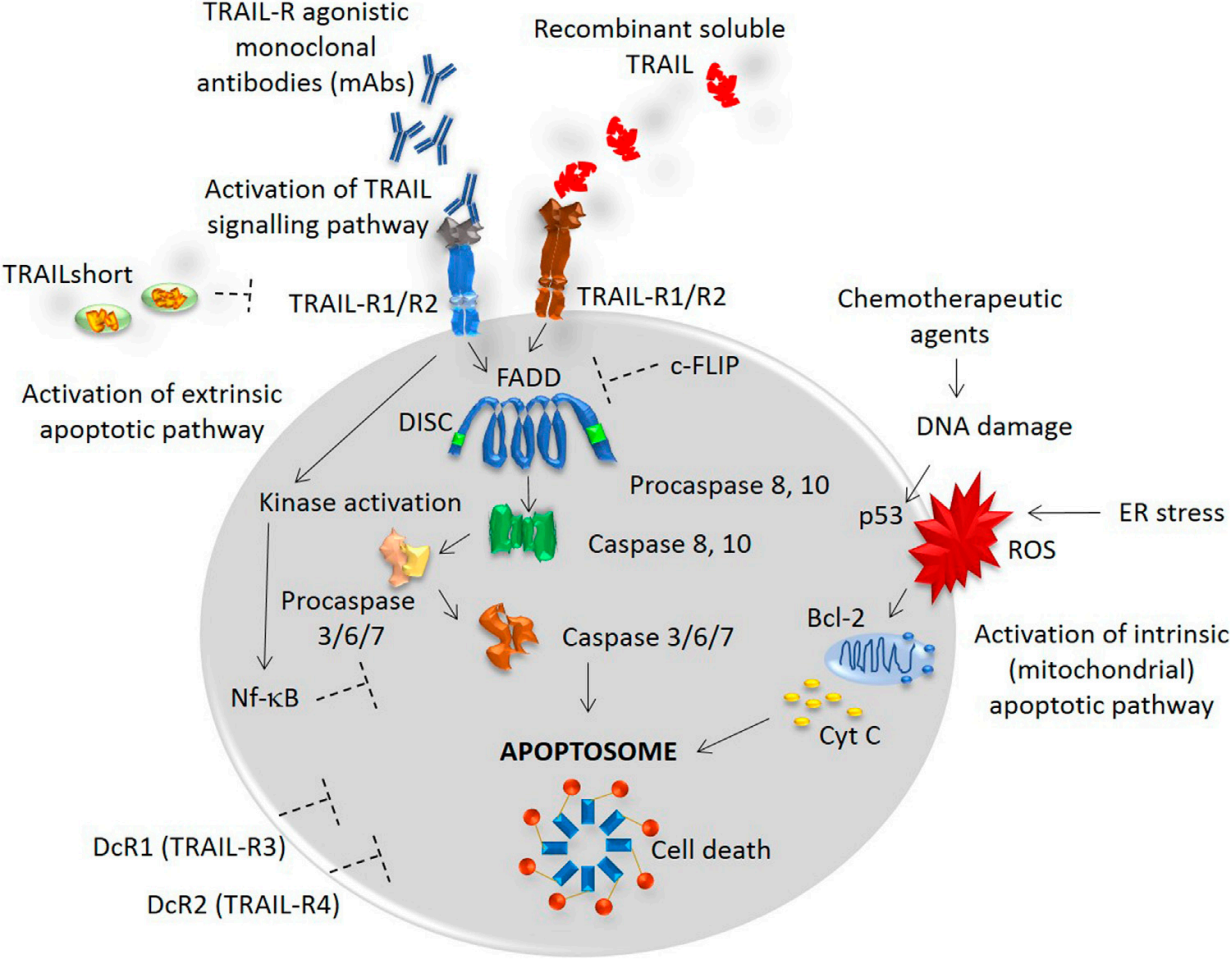
TRAIL signaling pathway. TRAIL binds to TRAIL-R1, R2, DcR1 and DcR2 receptors with a similarly high affinity. Death receptors TRAIL-R1 and TRAIL-R2 contain a conserved death domain (DD) motif and thus, binding of TRAIL transmits the apoptotic signal by causing a trimerization of the receptor and a formation of the death-inducing signaling complex (DISC). The recruitment of an adaptor molecule, FADD, is associated with the DISC formation and a subsequent binding and activation of caspases 8 and 10. This process allows the activation of other effector caspases, including the executor caspases, caspase-3 and caspase-7, triggering the final steps of apoptosis. TRAIL is known to trigger the extrinsic apoptotic pathway, while multiple other stimuli, such as chemotherapeutic agents or stimuli causing the DNA damage or ER stress, trigger the intrinsic (mitochondrial) apoptotic pathway. Depending on the cellular context, both intracellular inhibitors, such as c-FLIP and Nf-kB, and extracellular inhibitors, such as TRAILshort and DcR1/2 can restrain the caspase activation and contribute to the resistance to TRAIL-induced cell death.

TRAIL exists in two forms: the membrane-bound TRAIL (mTRAIL) and the soluble TRAIL (sTRAIL). Both forms can be found in different leukocyte subpopulations (Ehrlich et al., 2003). The binding of TRAIL to its death receptors, DR4 and DR5, causes a homotypic DD–dependent recruitment of Fas-associated protein with death domain (FADD). FADD bridges the pro–caspases 8 and 10 to form a death-inducing signaling complex (DISC), Figure 1. DISC further activates the caspase–8 which directly enables the activation of other effector caspases, including the executor caspases, caspase-3 and caspase-7 triggering the final steps of apoptosis (Gasparini et al., 2013). Since the apoptosis mediated by TRAIL is known to trigger the extrinsic signaling pathway, its combination with conventional chemotherapeutic drugs seems particularly relevant as they engage the intrinsic (mitochondrial) apoptotic pathway, Figure 1. To note, most stimuli induce apoptosis via the mitochondrial pathway (Lopez and Tait, 2015).

Within the cytoplasm, a number of inhibitors are recruited depending on the cellular context. The inhibitors, such as c-FLIP [cellular FLICE (FADD-like IL-1β-converting enzyme)-inhibitory protein], Bcl-2 (B-cell lymphoma 2), or DcR1/DcR2 can restrain the caspase activation and lead to the TRAIL-mediated apoptosis resistance, Figure 1 (Dubuisson and Micheau, 2017).

The mTRAIL and sTRAIL have been reported to recruit multiple procaspase molecules leading to the tumor cell death *in vitro* and *in vivo* (Ehrlich et al., 2003). Interestingly, it has been also revealed that signaling through FADD and DR5 (TRAIL-R2) serves as the key mechanism of chimeric antigen receptor (CAR) T cell cytotoxicity (Dufva et al., 2020). The ability of TRAIL to promote pro-tumorigenic activity against tumor cells regardless of their p53 status is thought to be the major advantage of TRAIL-based therapies (Dubuisson and Micheau, 2017).

The exception, however, is represented by KRAS-mutated cancers where TRAIL signaling was newly found to mediate migration, invasion, and metastasis. Even though studies have already proven that recombinant human TRAIL enhances the tumor sensitivity to chemotherapy/targeted therapy/radiotherapy in various cancer types, this may not apply to KRAS-mutated cancers (Yuan et al., 2018; von Karstedt and Walczak, 2020; Pal et al., 2016). KRAS-mutated cancers express high levels of TRAIL with the ability to promote tumor growth. Also, the activation of NF-κB signaling pathway by KRAS was found to interfere with the pro-apoptotic abilities of TRAIL-induced signaling (Wajant, 2004; Yang et al., 2016).

Nevertheless, it has been demonstrated that the resistance of KRAS-mutated cancers to the induction of apoptosis by exogenous TRAIL can be overcome by inhibiting the endogenous TRAIL (von Karstedt and Walczak, 2020). The therapeutic targeting of endogenous TRAIL signaling, thus, opens novel perspectives in the treatment options of KRAS-mutated cancers (von Karstedt and Walczak, 2020).

In parallel, a novel splice variant of TRAIL, named TRAILshort, was recently reported to antagonize the pro-apoptotic effects of TRAIL (Aboulnasr et al., 2020). The TRAILshort can be found in extracellular vesicles and serves as a dominant negative ligand for DR4 and DR5, Figure 1 (Aboulnasr et al., 2020).

In the following text, we will present the current status of all TRAIL-based monotherapies and combination therapies that have reached phase II and phase III clinical trials in humans. We will also discuss the novel TRAIL derivates and modifications that may entry clinical trials in the nearest future.

## TRAIL-R Agonistic Antibodies

TRAIL-R agonistic monoclonal antibodies bind specifically to either TRAIL-R1 (DR4) or TRAIL-R2 (DR5) with high affinity. The mechanism of action via TRAIL-R agonistic antibodies has been shown in preclinical models to induce also the antibody-dependent cellular cytotoxicity (ADCC) and complement-dependent cytolysis (CDC) (Qiu et al., 2012; Dai et al., 2015). However, to date, a single-phase III study has not been carried out (clinicaltrials.gov).

### TRAIL-R1 Agonistic Antibodies

While different agonistic TRAIL-R1 antibodies have been developed, such as HS101, 4HS, or 4G7, only HGS-ETR1 antibody, also known as mapatumumab, has entered clinical trials in humans and has reached the second phase of clinical testing (Dai et al., 2015; Medler et al., 2019; Chuntharapai et al., 2001). Mapatumumab was reported to be well tolerated up to 20 mg/kg daily and has already been tested in the treatment of non-small cell lung carcinoma (NSCLC), multiple myeloma, non-Hodgkin’s lymphoma, and hepatocellular carcinoma, Table 1. In phase I, as well as in phase II clinical trials, mapatumumab has shown a great safety profile and, furthermore, caused complete or partial clinical responses when administered as monotherapy in patients with follicular non—Hodgkin’s lymphoma (Younes et al., 2010). The stability of the disease was achieved in 30% of the patients (Younes et al., 2010). The efficacy of mapatumumab, however, was not supported by other phase II clinical trials, and to date, phase III clinical trials with mapatumumab have not yet been initiated.

**TABLE 1:** Phase II and III clinical trials with TRAIL-R agonistic antibodies, multivalent antibodies, recombinant TRAIL, and TRAIL derivates and modifications.

Drug name	Diagnosis	Mechanism of action	Trial design	Setting	Dosage regimen	Nr. of participants	Study completion	ID
Trail-R agonistic antibodies: phase II clinical trials								
Mapatumumab	Advanced hepatocellular carcinoma	Monoclonal antibody targeting TRAIL-R1	A randomized, multi-center, blinded, placebo-controlled study	Combination therapy (Sorafenib, First-line)	30 mg/kg i.v. on day 1 of each 21-days cycle	101 pts. (>18 yrs.)	November 2017	NCT01258608
Mapatumumab	Multiple myeloma	Monoclonal antibody targeting TRAIL-R1	Multi-center, open-label, randomized study	Combination therapy (Bortezomib)	10 mg/kg i.v. on day 1 of each 21-days cycle	105 pts. (>18 yrs.)	October 2010	NCT00315757
Mapatumumab	Relapsed or refractory non-hodgkin’s lymphoma	Monoclonal antibody targeting TRAIL-R1	A multi-center, open-label, dose-escalation study	Monotherapy	10 mg/kg i.v. on day 1 of each 21-days cycle	40 pts. (>18 yrs.)	May 2007	NCT00094848
Mapatumumab	Advanced non-small cell lung cancer	Monoclonal antibody targeting TRAIL-R1	Randomized, Multi-Center, Open-Label Study	Combination therapy (Carboplatin, Paclitaxel), First-line	10 mg/kg i.v. on day 1 of each 21-days cycle	111 pts. (>18 yrs.)	February 2011	NCT00583830
Tigatuzumab	Metastatic or unresectable non-small cell lung cancer	Monoclonal antibody targeting TRAIL-R2	Randomized, double-blinded, placebo controlled	Combination therapy (Carboplatin/Paclitaxel)	10 mg/kg	109 pts. (>18 yrs.)	December 2011	NCT00991796
Tigatuzumab	Pancreatic Cancer	Monoclonal antibody targeting TRAIL-R2	Phase 2 multicenter, open-label study	Combination therapy (Gemcitabine)	8 mg/kg loading dose followed by 3 mg/kg weekly	65 pts. (>18 yrs.)	December 2008	NCT00521404
Tigatuzumab	Metastatic triple negative breast cancer	Monoclonal antibody targeting TRAIL-R2	An open label, randomized study	Combination therapy (Abraxane)	10 mg/kg loading dose; 5 mg/kg for the 1st cycle, than every other week on Days 1 and 15 for subsequent cycles	64 pts. (>18 yrs.)	June 2017	NCT01307891
Conatumumab	Pancreatic cancer	Monoclonal antibody targeting TRAIL-R2	Randomized, double-blind study	Combination therapy (Gemcitabine)	10 mg/kg	138 pts. (>18 yrs.)	April 2012	NCT00630552
Multivalent antibodies: phase II clinical trials								
Gen1029	Colorectal cancer non-small cell lung cancer triple negative breast cancer renal cell carcinoma gastric cancer pancreatic cancer	1:1 mixture of two humanized non-competing DR5-specific mAbs, each carrying an E430G hexamerization-enhancing mutation	Randomized, open-label, multicenter study	Monotherapy	Intravenously once every 14 days, starting dose + escalation steps	520 pts. (>18 yrs.)	Estimated March 2022	NCT03576131
Recombinant TRAIL: phase II clinical trials								
Dulanermin	B-Cell non-hodgkin’s lymphomas that have progressed following previous rituximab therapy	Recombinant TRAIL triggering apoptosis via activation of DR4 and DR5	Randomized, open-label, multicenter study	Combination therapy (Rituximab)	8.0 mg/kg/day dose i.v. for 5 consecutive days at the start of each 21-days cycle	72 pts. (>18 yrs.)	May 2010	NCT01258608
Dulanermin	Previously untreated stage IIIb/IV non-small cell lung cancer (NSCLC)	Recombinant TRAIL triggering apoptosis via activation of DR4 and DR5	A multicenter, open label, randomized study	Combination therapy (Bevacizumab, Paclitaxel, Carboplatin), First-line	20 mg/kg on days 1–2 per 21 days cycle	213 pts. (>18 yrs.)	November 2011	NCT00315757
Recombinant TRAIL: phase III clinical trials								
Dulanermin	Advanced non-small cell lung cancer	Recombinant TRAIL triggering apoptosis via activation of DR4 and DR5	A randomized, double-blind, placebo-controlled study	Monotherapy	150 μg/kg/d IV (in the vein), on day 1 to 7 of each 21 days cycle	417 pts. (>18 yrs.)	June 2018	NCT00583830
Trail derivates and modifications: phase II clinical trials								
MSCTRAIL	Non-small cell lung cancer (NSCLC)	Targeted stem cells expressing TRAIL	Multicentre, randomised double blind placebo controlled	Combination therapy (pemetrexed/cisplatin chemotherapy)	Not specified	46 pts. (>18 yrs.)	Estimated September 2025	NCT03298763
CPT	Relapsed and refractory multiple myeloma	Circularly permuted TRAIL triggering apoptosis via activation of DR4 and DR5	A multicenter, open label study	Combination therapy (Thalidomide)	5–10 mg/kg; thalidomide 100 mg	43 pts. (>18 yrs.)	2014	ChiCTRONC-1200206

The efficacy of combination therapies with mapatumumab was assessed throughout various malignancies (Micheau et al., 2013). Most of the combinations, such as mapatumumab with paclitaxel, gemcitabine, carboplatin or bortezomib, however, did not improve the response rate compared to each compound alone (Micheau et al., 2013).

### TRAIL-R2 Agonistic Antibodies

So far, several clinical trials with agonistic TRAIL-R2 antibodies have been carried out. Among TRAIL-R2 targeting agents, zaptuzumab, KMTR2, or DJR2 have not yet been tested in humans (Pal et al., 2016; Aboulnasr et al., 2020; Qiu et al., 2012). Both zaptuzumab and KMTR2 have, however, shown the ability to trigger apoptosis in the absence of TRAIL-R2 crosslinking (Dubuisson and Micheau, 2017). Lexatumumab, drozitumab, and LBY-135, have completed the phase I clinical trials, and tigatuzumab and conatumumab entered the phase II of clinical testing, Table 1. The targeted cancers were pancreatic cancer, breast cancer, and lung cancer. Even with the encouraging results with TRAIL agonists *in vitro*, tigatuzumab and conatumumab treatment results *in vivo* were rather disappointing (Cohn et al., 2013; Forero-Torres et al., 2013; Fuchs et al., 2013; Reck et al., 2013). Whereas both agents were well tolerated, the anticipated anti-cancer effect was not attained (Forero-Torres et al., 2013; Fuchs et al., 2013; Reck et al., 2013). In advanced NSCLC patients, tigatuzumab did not improve the efficacy of carboplatin/paclitaxel (Reck et al., 2013). In patients with metastatic pancreatic adenocarcinoma, the study group with conatumumab administration had a negligibly higher 6-month survival rate (59%) as compared to the placebo (50%) (Kindler et al., 2012). Interestingly, conatumumab was found to decrease the OR rate of doxorubicin and the results from clinical trials evaluating conatumumab with other pharmacological agents, such as paclitaxel, carboplatin and panitumumab, have not been divulged yet (NCT00630786, NCT00534027).

Of note is a promising phase I clinical trial with a novel TRAIL-R2 antibody DS-8273a which was terminated in 2017 for non-specified reasons. In this study, four colorectal carcinoma patients were administered the DS-8273a in a combination with anti-PD-1 agent nivolumab. The results were not published (NCT02991196). Currently, an open-label, multicenter, phase I study with a novel recombinant humanized tetravalent antibody targeting TRAIL-R2 is recruiting patients with locally advanced or metastatic solid tumors, including sarcomas (NCT03715933).

### Novel Multivalent-Based Antibodies

The administration of conventional TRAIL-R1/R2 agonistic antibodies has not yet resulted in an improved survival of cancer patients in clinical trials. It is presumed that the main hurdle lies in an insufficient capacity of these agents to induce TRAIL-R clustering which is essential for the apoptosis induction (Dubuisson and Micheau, 2017). For that reason, multivalent anti-DR4 (TRAIL-R1) and anti-DR5 (TRAIL-R2) antibodies were designed and almost instantly showed superior efficacy as compared to monovalent and divalent antibodies (Dubuisson and Micheau, 2017). A tetravalent sc-Fv:DR5 derivate and a single-chain scFv:DR5 nanobody represented the most promising agents in preclinical models, however, the scFv:DR5 nanobody was associated with hepatotoxicity in phase I. clinical trials (Liu et al., 2015; Papadopoulos et al., 2015) Another promising agent, a DR4/DR5 dual-specific Kringle domain, is expected to enter clinical trials due to its ability to induce TRAIL– and reactive oxygen species (ROS)-mediated cell death (Jeong et al., 2014). Moreover, other bi-DR4/5 specific antibodies are currently being developed (Dobson et al., 2009). To date, more phase I clinical trials are currently ongoing with DR5-specific HexaBody molecules (NCT03576131), a tetravalent anti-DR5 INBRX-109 (NCT03715933) or with a multivalent IgM DR5 agonist (NCT04553692) for the treatment of solid tumors. The approach to design an antibody cross-reactive to both DR4 and DR5 displayed only limited therapeutic potential *in vitro* (Dubuisson and Micheau, 2017). Bi-specific antibodies were tested across various malignancies and promising preclinical results were achieved with MCSP (melanoma-associated chondroitin sulfate proteoglycan) and DR5 bispecific, tetravalent antibody which allowed a selective DR5-dependent apoptosis induction in the MCSP-expressing melanoma cells (Dobson et al., 2009; He et al., 2016; Dubuisson and Micheau, 2017).

## Recombinant TRAIL

The mechanism of action of recombinant soluble TRAIL protein is based on its binding to TRAIL-R1 and TRAIL-R2 leading to subsequent activation of caspases and the apoptotic cell death (Zhang et al., 2010). The main limitation of the systematically applied soluble TRAIL appears to be in its rapid clearance from the serum (Lemke et al., 2014). The results with recombinant TRAIL, however, are so far encouraging, Table 1.

### Dulanermin

Dulanermin is the first soluble recombinant version of the naturally occurring human protein TRAIL (Quintavalle and Condorelli, 2012). Dulanermin has reached phase II in patients with NSCLC and B-cell lymphomas (Soria et al., 2011; Cheah et al., 2015). A phase III clinical trial with 452 stage IIIB and stage IV NSCLC patients as monotherapy has been completed in 2018 (NCT03083743). In this randomized, placebo-controlled clinical trial, the patients were randomly assigned to receive either dulanermin plus chemotherapy (vinorelbine and cisplatin) or placebo plus chemotherapy. In this study, the addition of dulanermin to chemotherapy in patients with untreated advanced NSCLC improved PFS (6.4 months vs. 3.5 months in the placebo arm). Moreover, the results applied for all patients in the dulanermin arm and were consistent across all clinical subgroups (Ouyang et al., 2018). This synergic potential of dulanermin was also reflected in the increased objective response rate (ORR). In addition, the incidence of adverse events did not differ between the two study arms. At the time of analysis, however, the OS was not improved in the dunalermin arm as compared to the placebo arm (Ouyang et al., 2018). Another recombinant TRAIL variant which has entered the clinical trials was circularly permuted TRAIL, also known as CPT (Micheau et al., 2013). Early clinical trials showed great tolerability with CPT alone or in combination therapy (Micheau et al., 2013). Moreover, CPT exhibited more robust pro-apoptotic activity than dulanermin (Fang et al., 2005). However, patients who were enrolled in the second phases of clinical testing experienced mostly partial responses, whereas novel severe side effects were reported, such as CPT-mediated liver injury (Chen et al., 2012).

### SCB-313

SCB-313 is a recombinant fully-human TRAIL-trimer fusion protein administered to patients for the first time at the end of 2019 (NCT04123886). Five clinical trials with SCB-313 are currently recruiting patients with the following indications: malignant ascites, peritoneal carcinomatosis, and malignant pleural effusions (clinicaltrials.gov). The efficacy and safety of SCB-313, therefore, remains to be determined.

### TRAIL Derivates

Several TRAIL derivates have been designed to allow better target specificity. Single-chain variable fragments of TRAIL (scFv-TRAIL) were described to increase the tumor-killing efficiency and were initially divided into two major groups. The first group of molecules allows dual targeting by construction of scFv-TRAIL associated with a tumor-associated antigen, such as epidermal growth factor (EGFR), multidrug resistance protein (MRP3), mesothelin and others (Dubuisson and Micheau, 2017). The second group of scFvs molecules represents recombinant proteins with the ability to target immune cell antigens, such as CD25, CD47, PD-L1 and multiple others (Dubuisson and Micheau, 2017).

In the case of scFv-PD-L1:TRAIL derivate, the TRAIL-mediated apoptosis is accompanied by a checkpoint inhibition leading to an increased T cell activation (Hendriks et al., 2016). Novel TRAIL derivates are very promising and clinical trials are urgently needed.

### TRAIL-Conjugates, Fusion Proteins and Genetically Engineered Modifications

To overcome TRAIL resistance, several attempts to conjugate TRAIL with cytotoxic drugs were carried out. So far, a TRAIL conjugate with monomethyl auristatin E (MMAE), the TRAIL-MMAE, was shown to deliver a potent antitumor response in breast carcinoma xenograft animal models (Pan et al., 2015). Moreover, the TRAIL-MMAE exhibited great pharmacokinetic abilities with minimal toxicity (Pan et al., 2015). Encouraging results were also observed in animal models treated with AD-053.2, a TRAIL conjugate with mitochondrial protein Smac/DIABLO which potentiates different forms of apoptosis (Pieczykolan et al., 2014).

Recently, a fusion protein ABBV-621 composed of three TRAIL receptor binding domains and an Fc domain of a human immunoglobulin G (IgG) entered a phase I clinical trial both in monotherapy and in combination therapies (NTC03082209). Multiple study arms evaluated the ABBV-621 efficacy in the treatment of solid or hematological malignancies (Ratain et al., 2019). The preliminary results released by the investigators suggested mostly the capability of ABBV-621 to maintain a disease stability (Calvo et al., 2019; Ratain et al., 2019).

Another novel approach with targeted stem cells expressing TRAIL, MSCTRAIL, has been introduced in a study initiated in 2019 (NCT03298763). The results of the second phase are eagerly awaited.

Chimeric antigen receptor (CAR) T cell therapy has revolutionized the treatment of advanced B cell leukaemias and lymphomas (Pehlivan et al., 2018; Mika et al., 2020). This novel approach of genetically engineered T cells allows T cells to bind in an antibody-like manner without MHC molecules, which provides cancer specificity and a high-binding efficacy (Feins et al., 2019). To date, CAR T cells targeting TRAIL receptors were profoundly discussed, however, only a single agent, TR1-svFv-CAR was constructed and evaluated in an *in vitro* setting (Kobayashi et al., 2014). In this model, TR1-svFv-CAR exhibited cytolytic activity toward DR4-deficient Jurkat cells, peripheral blood cells and NK cell lines (Kobayashi et al., 2014).

## Combination Strategies

It has been reported that some cancers are TRAIL-therapy resistant for various reasons (Qiu et al., 2012; Dai et al., 2015; Wajant, 2019). In such cases, a combination strategy may provide a rationale for sensitizing the tumor microenvironment to TRAIL-therapies, and either chemotherapy or different therapeutic approach may serve as the pharmacological modulator.

### Chemotherapy and Radiotherapy

Both TRAIL agonistic antibodies and recombinant TRAIL have proven good safety profiles and, therefore, can be easily combined with chemotherapy and/or radiotherapy.

In cervical cancer cell lines, mapatumumab in combination with irradiation enhanced the cell apoptosis from 51to 83%, compared to mapatumumab alone (NCT01088347). The initial studies indicated that even though some tumors are primary TRAIL-resistant, chemotherapy or radiotherapy may overcome the resistance (Quintavalle and Condorelli, 2012). This was partially supported by a recent study, where the addition of dulanermin to chemotherapy provided superior clinical benefits for patients with advanced NSCLC (Ouyang et al., 2018).

### Immunotherapy, Biological Therapy and Targeted Therapy

Immunotherapy, biological therapy, and targeted therapy have significantly improved the OS in various malignancies and were shown to largely affect the tumor microenvironment (Schirrmacher, 2019; Hirata and Sahai, 2017). Therefore, these approaches may support the TRAIL-based therapies by overcoming the tumor cell resistance to TRAIL. The synergistic effect of targeted therapy has been observed in mapatumumab studies (NCT01258608, NCT00315757), whereas biological therapy was mostly applied in combinations with dulanermin (NCT00400764, NCT00508625). A single-phase I trial with a monoclonal antibody targeting TRAIL-R2 administered together with checkpoint immunotherapy was terminated in 2017 (NCT02991196).

## Modulation of the TRAIL Expression and Signaling

Researchers highlighted that the activation of the TRAIL apoptotic pathway could be achieved by administering TRAIL therapy together with other agents increasing the expression of TRAIL and/or its receptors DR4 and DR5 (von Karstedt and Walczak, 2020; Dai et al., 2015; Wajant, 2019). As it is known that TRAIL signaling pathway is shaped by the Nf-κB signaling pathway, the modulation of the Nf-κB signaling was used to affect the TRAIL responses *in vivo* and to sensitize the tumor to TRAIL-induced apoptosis. For that purpose, sulforaphane, melittin, resveratrol, wogonin, and multiple other components were tested (Dai et al., 2015). Six clinical trials are currently ongoing and/or recruiting with the use of sulforaphane in the treatment of cancer (clinicaltrials.gov).

## Discussion

TRAIL and its death receptors TRAIL-R1 and TRAIL-R2 trigger the apoptotic cell death in tumor cells without damaging non-malignant cells. This phenomenon has been illustrated in a number of *in vitro* and *in vivo* studies (Yuan et al., 2018; von Karstedt & Walczak, 2020; Dai et al., 2015; de Miguel et al., 2016).

The mechanism underlying the selective induction of apoptosis in tumor cells has not yet been elucidated, even though the significantly higher expression of TRAIL’s decoy receptors in non-malignant cells may provide some answers (Micheau et al., 2013).

Because the gene encoding protein p53 is often mutated in cancers, TRAIL's ability to activate apoptosis independently of the p53, together with its tumor-selectivity, made TRAIL an attractive therapeutic target (Elmore, 2007; Lim et al., 2019). Yet, owing to TRAIL's ability to bind to four TRAIL receptors with a similarly high affinity, the understanding of TRAIL signaling regulation represents a grand challenge (Micheau et al., 2013). What creates even more confusion is that selective engagement of either TRAIL-R1 or TRAIL-R2 occurs in diverse cancer types (MacFarlane et al., 2005; Natoni et al., 2007; Surget et al., 2012). Moreover, the expression of TRAIL receptors is not known in a significant proportion of malignancies and since it has been already demonstrated that the TRAIL-Rs expression differs among different cancers, it might be presumed that the expression can also vary among individuals with the same cancer type (Min et al., 2004; McCarthy et al., 2005).

In spite of the promising preclinical observations, the TRAIL-based therapies in humans have certain limitations. The TRAIL-R’s agonistic antibodies seem to rather elicit a limited agonistic potential, and only a few drugs have entered the phase II clinical trials. The main reason for the clinical failure of the first generation of antibodies was their weak ability to induce the receptor aggregation which is necessary for triggering the downstream apoptotic pathway (Dubuisson and Micheau, 2017). Multivalent antibodies seemed to overcome this challenge. However, these antibodies also elicited many adverse events (Liu et al., 2015; Papadopoulos et al., 2015). So far, satisfactory results with agonistic antibodies were attained in follicular non-Hodgkin’s lymphoma, where the stability of the disease was achieved in 30% of the patients receiving mapatumumab (Younes et al., 2010).

The antitumor activity of soluble TRAIL agents was mostly seen in the treatment of NSCLC, where the recombinant protein dulanermin has reached clinical phase III (Ouyang et al., 2018). It is presumed that the favorable safety profile of dulanermin along with its so far limited potency to improve the OS will promote further studies of this agent in different settings and therapeutic combinations (Ouyang et al., 2018). Interestingly, it has been demonstrated that concomitant administration of chemotherapy and recombinant TRAIL may restore sensitivity to TRAIL-induced apoptosis in primary TRAIL-resistant tumor cells (Micheau et al., 2013).

Therefore, to understand mechanisms that drive the expression of TRAIL-Rs, the extensive analysis of the expression status prior- and post- chemotherapy/targeted therapy/immunotherapy should be conducted.

The so-far modest efficacy of the TRAIL-agonistic antibodies and recombinant TRAIL, the therapies could still show unexpected efficacy upon combining with other treatment approaches that promise to condition the cancer cells to the TRAIL-induced apoptosis.
